# Successful Removal of Intra-Uterine Tumor

**Published:** 1866-02

**Authors:** John Meacham

**Affiliations:** Racine, Wis.


ART. V.—*Successful Removal of Intra-Uterine Tumor.* BY JOHN MEACHAM, M. D., *Racine, Wis.*

I was called in October last to visit Mrs. Bankers, of Lyons, Walworth county, in this State, who had been five years suffering from the presence of an immense uterine tumor, which reached as hign as the umbilicus. I found by a careful examination with the uterine sound that the tumor had its attachment at the very fundus of the organ, for I could pass the instrument completely around the morbid growth to within two or three inches of that portion of the uterus. The os was not large, but sufficiently so to enable me to make the examination, and arrive at the conclusions stated above. She was very anxious to rid herself of her long carried burden, and strongly urged me to undertake an operation for its removal, if I thought there was a possible chance of her surviving it. By the speculum I could get a very excellent view of the portion of the tumor that presented at the mouth of the womb. It had a light and shining surface, like fibrous tissue, and was hard and unyielding to pressure like that structure. Her constitution had very much given way from the long continued presence of the tumor. She was obliged to evacuate the bladder every few minutes, from the pressure of the morbid growth upon it; the bowels scarce ever moved without the aid of laxatives; the limbs were œdematous. She was greatly emaciated; the countenance very sallow, and her general appearance indicated far advanced malignant disease. My opinion, however, was that the tumor was fibrous in character, and not malignant, and that an operation might prove successful, and that the only possible way to remove it was by ligature. Dr. Darling, of Burlington, who was with me at the time, and whose patient she had been, concurred with me in opinion.

I procured at the instrument-makers a long, curved, double canula, so arranged that the tubes could be detached from the fastenings which held them together, and separated the one from the other. These were now armed with a very strong and hard twisted, silken cord, passing up one tube, down through the other, and projecting sixteen inches from the end of either. The tubes were now passed together into the womb, along its floor, until it reached the inferior and posterior attachment of the tumor. They were then separated, and one tube brought over to the superior surface of the tumor, on one side, and the other upon the other side, in the same manner, until they approximated. It was now found that the circumference of the tumor, at the point encircled by the ligature, was eighteen inches. This was readily told by the amount of the silk cord used in surrounding it. A double slide was now passed over the tubes, binding them strongly together, and one end of the cord securely fastened to an eye attached to the slide. Very firm traction was now made upon the cord, as its strength would warrant, and it was firmly secured. A large anodyne was given, and the patient allowed to rest for twenty-four hours; at the end of which time the cord was again powerfully drawn upon, when it was ascertained that a progress of three inches had been made towards cutting off the tumor. This process was continued for eleven successive days, when the canula and cord came away, and soon after by a violent contraction of the uterus the whole dead mass was expelled. There had been slight uterine efforts for three or four days prior to its final separation, accompanied by a very fetid discharge. The weight of the tumor after these eleven days of strangulation was nine pounds, and its hardened structure was as apparent as at the day of ligation.

The patient has been improving rapidly since, and only regrets that she had not been operated upon before.

---

ON THE TREATMENT OF GUN-SHOT WOUNDS.—With reference to Dr. Smart's letter on the plan, to which he ascribes novelty, of paring the edges of gun-shot wounds, converting them into simple incised wounds, and thus aiming at union by first intention, it may be of interest to remind your readers that this treatment was used and described by Larrey as applicable to gnn-shot wounds of the face. The references are: "Memories de Chirurgie Militaire," tome iii, p. 258, et tome iv, p. 240.

JOSEPH BELL, F. R. C. S;

*London Lancet, April,* 1865.

